# Supplementary material for: Ancestry-specific gene expression in peripheral monocytes mediates risk of neurodegenerative disease
Source: bioRxiv. 2024 Nov 22:2024.11.20.624489. Preprint. [Version 1] doi: 10.1101/2024.11.20.624489 (PMC11722246; doi:10.1101/2024.11.20.624489)
Supplement: Supplement 2 [file media-2.pdf]

Supplementary information

Ancestry-specific gene expression in peripheral monocytes mediates risk of neurodegenerative disease

Supplementary Tables

Supplementary Table 1

Disease	Gene
AD	ABCA7
AD	ABI3
AD	ADAM10
AD	APOE
AD	APP
AD	BIN1
AD	CD33
AD	CR1
AD	NOTCH3
AD	PLCG2
AD	PLD3
AD	PRNP
AD	PSEN1
AD	PSEN2
AD	SORL1
AD	SPI1
AD	TREM2
AD	UNC5C
PD	ATP13A2
PD	DNAJC6
PD	FBXO7
PD	GBA
PD	LRRK2
PD	PARK7
PD	PINK1
PD	PLA2G6
PD	POLG
PD	PRKN
PD	RAB32
PD	SNCA
PD	SYNJ1
PD	VPS13C
PD	VPS35
FTD	C9orf72
FTD	FUS
FTD	GRN
FTD	MAPT
FTD	TARDBP
FTD	TBK1

Supplementary Table 1: Table of genes causally implicated in Neurodegenerative diseases. Alzheimer's disease (AD) genes were derived from Neuner et al[1], Parkinson's disease (PD) genes from Blauwendraat et al[2], and Frontotemporal dementia (FTD) genes from Antonioni et al[3].

Supplementary Table 9

Data Resource	Link	Notes
Antonioni et al[3]	https://doi.org/10.3390/ijms241411732	Data taken from tables and supplementary tables.
Aquino et al[4]	https://doi.org/10.1038/s41586-023-06422-9	Data in Figure 1 taken from tables and supplementary tables. Data for Figure 2 downloaded from https://dataset.owey.io/doi/10.48802/owey.e4qn-9190 on 6th April 2024.
Blauwendraat et al[2]	https://doi.org/10.1016/S1474-4422(19)30287-X	Data taken from tables and supplementary tables.
Chen et al[5]	https://doi.org/10.1016/j.cell.2020.06.045	Downloaded using the opentargets portal on 9th September 2024.
Foo et al[6]	https://doi.org/10.1001/jamaneurol.2020.0428	Summary statistics provided by authors.
Jansen et al[7]	https://doi.org/10.1038/s41588-018-0311-9	Summary statistics downloaded from https://cncr.nl/research/summary_statistics/ on 12th May 2024.
Nalls et al[8]	https://doi.org/10.1016/S1474-4422(19)30320-5	Downloaded from https://pdgenetics.org/resources, excluding 23andMe cases.
Nedelec et al[9]	https://doi.org/10.1016/j.cell.2016.09.025	QTD000379.all.tsv.gz, QTD000384.all.tsv.gz and QTD000389.all.tsv.gz downloaded from https://www.ebi.ac.uk/eqtl/ on 13th March 2024.
Neuner et al[1]	https://doi.org/10.1016/j.nbd.2020.104976	Data taken from tables and supplementary tables.
Ota et al[10]	https://doi.org/10.1016/j.cell.2021.03.056	Downloaded from https://ddbj.nig.ac.jp/public/ddbj_database/gea/experiment/E-GEAD-000/E-GEAD-420/ on 21st May 2024.
Quach et al[11]	https://doi.org/10.1016/j.cell.2016.09.024	QTD000409.all.tsv.gz, QTD000414.all.tsv.gz, QTD000419.all.tsv.gz, QTD000424.all.tsv.gz, and QTD000429.all.tsv.gz downloaded from https://www.ebi.ac.uk/eqtl/ on 13th March 2024.
Rizig et al[12]	https://doi.org/10.1101/2023.05.05.23289529	Summary statistics provided by authors.
Shigemizu et al[13]	https://doi.org/10.1038/s41398-021-01272-3	Summary statistics provided by authors.

Supplementary Table 9: List of datasets utilised in this study.

Supplementary Figures

Supplementary Figure 1

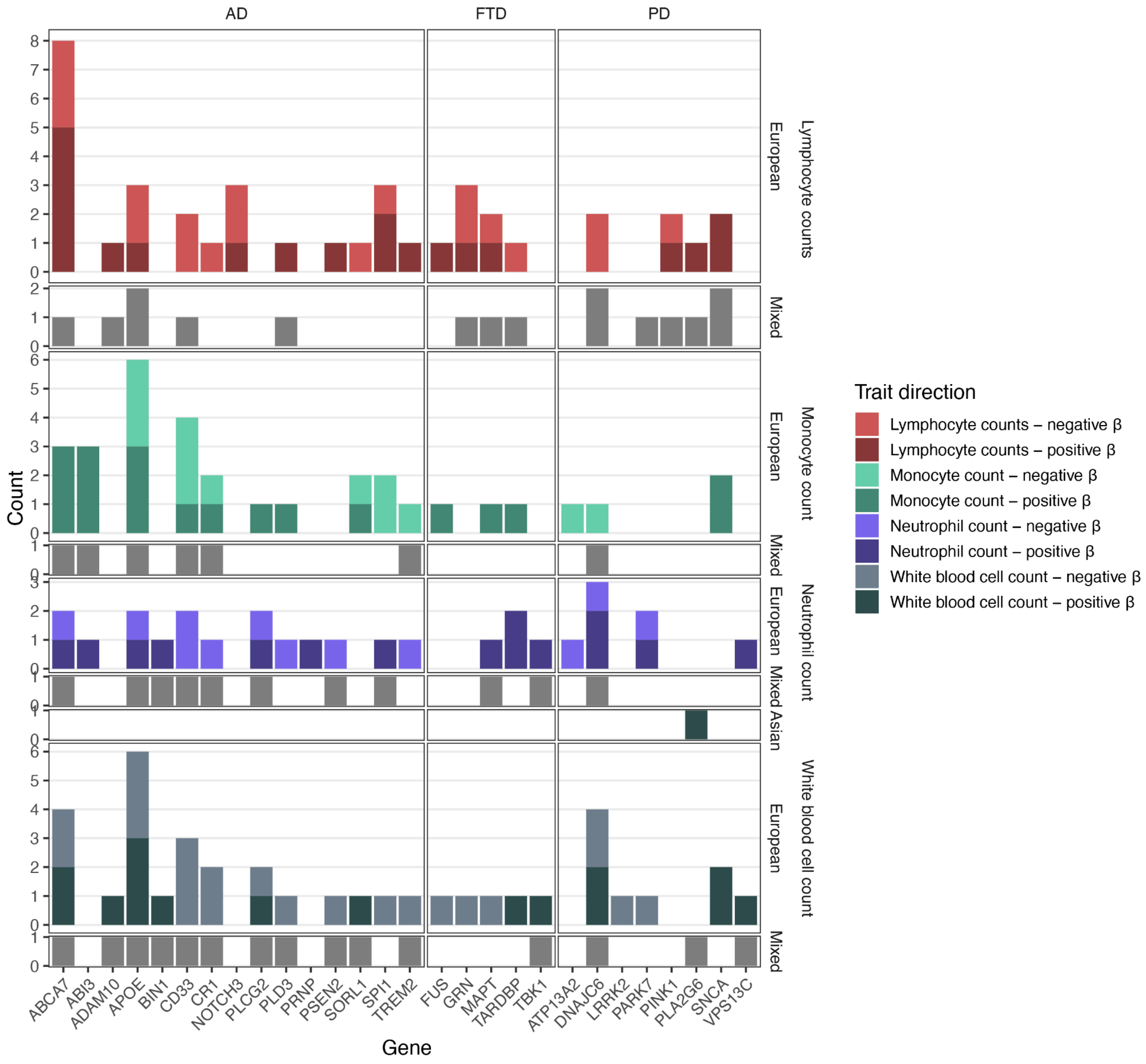

Supplementary Fig. 1: Phenotype QTL associations between NDD genes and white blood cell count metrics. Number of pQTL associations between NDD genes and 4 white blood cell count metrics from Chen et al[5] (top to bottom): lymphocyte, monocyte, neutrophil and total white blood cell counts. Barchart shows the count of ancestry-specific results and muti-ancestry meta-analysis. Ancestry-specific results were coloured by direction of effect (β), though in the multi-ancestry meta-analysis this was not reported.

Supplementary Figure 2

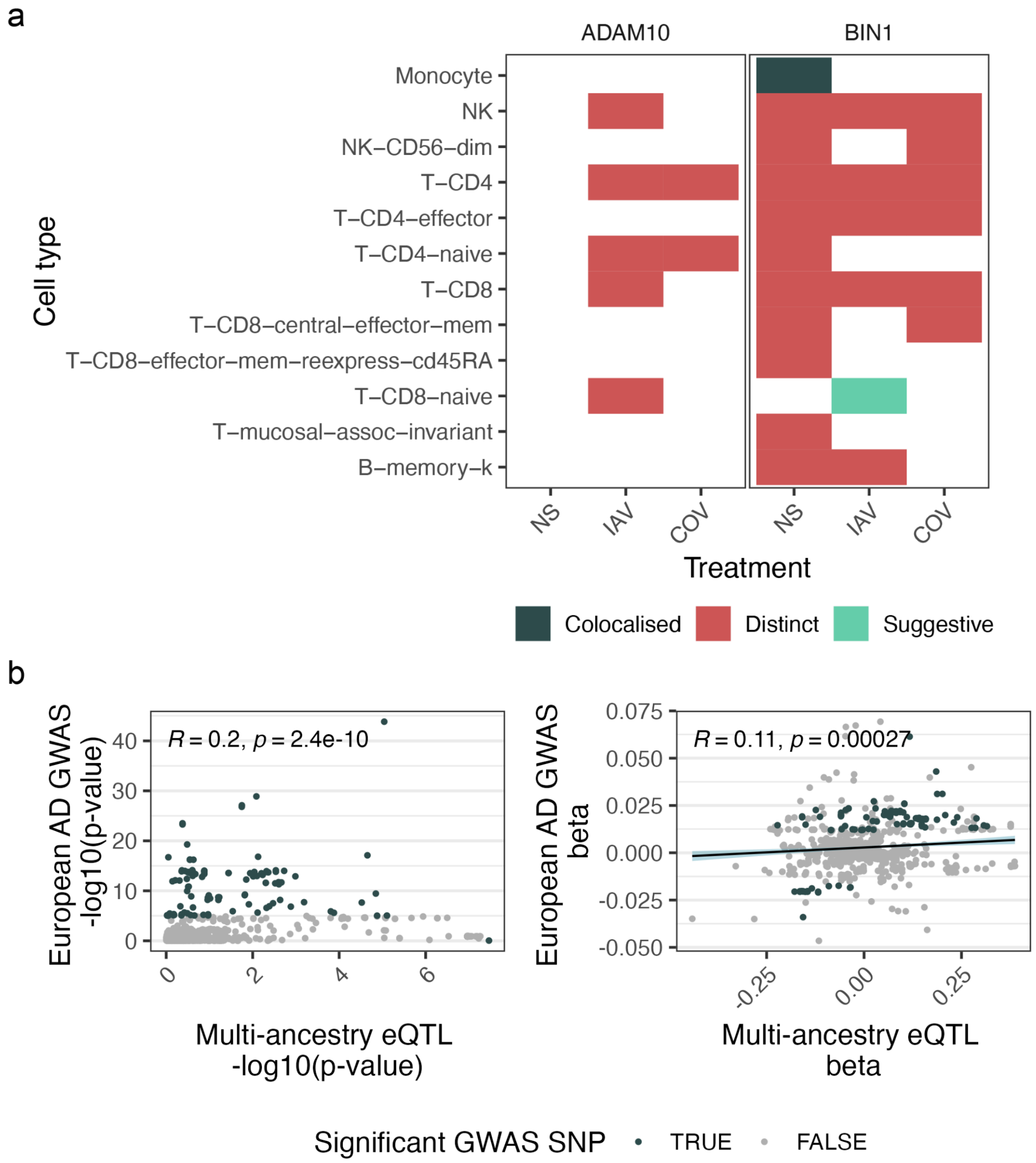

Supplementary Fig 2. Colocalisation results of AD-causing genes in European AD GWAS and Multi-ancestry eQTL. a, Results for genes causally implicated in AD across cell types and activation states. Colocalisation defined by PPH4 > 0.75; Distinct is defined by PPH3 > 0.75; Suggestive is defined where the ratio of PPH4/PPH3 is high (ratio_PPH4_PPH3 > $\log_2(9)$), but there is insufficient power to conclude a significant colocalisation (PPH3 + PPH4 is between 0.5-0.75). NS, no stimulation; IAV, influenza A virus; COV, SARS-CoV-2 virus). **b**, Locus analysis of significant colocalising signal at BIN1 in unstimulated monocytes,, showing p-values at left, and betas at right. Significant GWAS SNP defined by -log10(p-value) > 5×10^{-8}, inset text shows Pearson’s correlation coefficient and p-value, and beta plot also show regression line of best fit and 95% confidence intervals.

Supplementary Figure 3

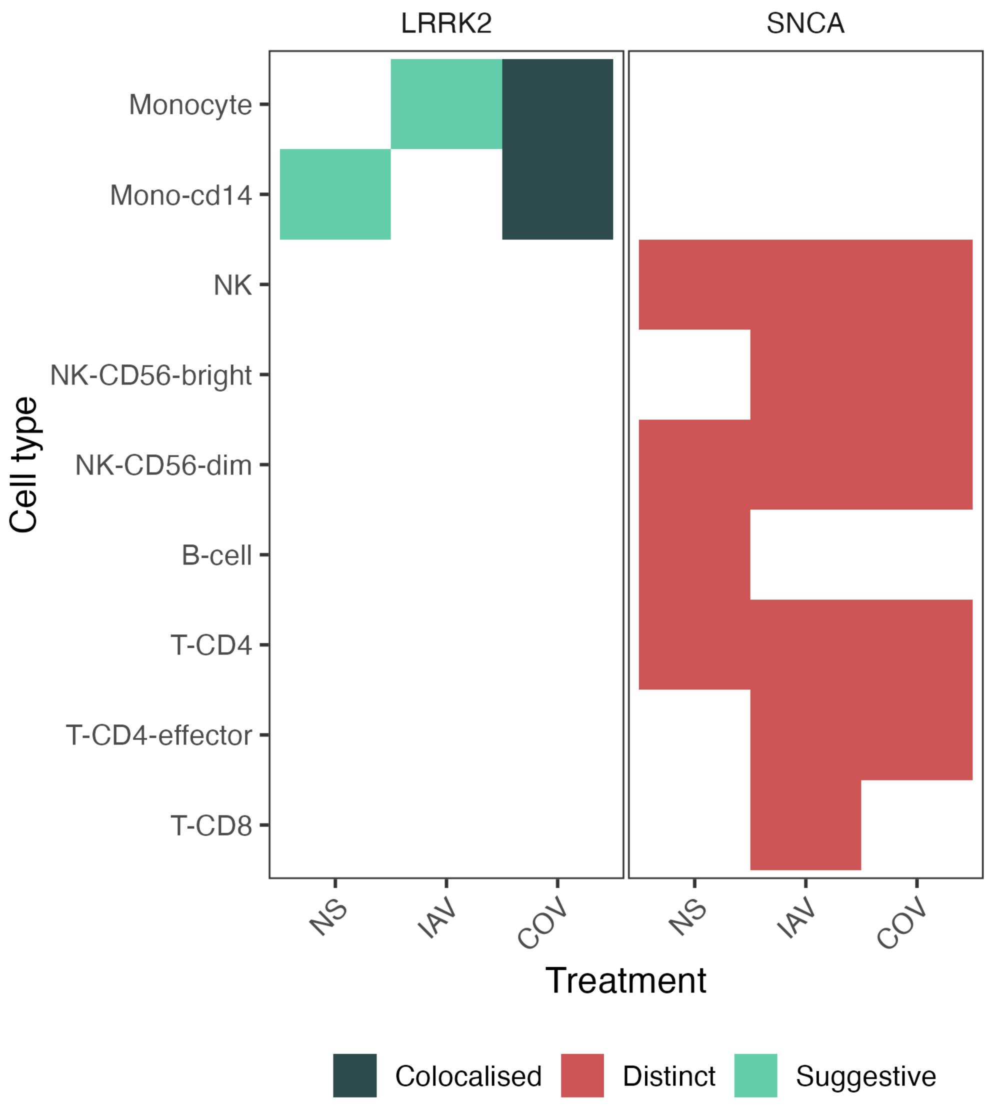

Supplementary Fig 3. Colocalisation results in European PD GWAS and Multi-ancestry eQTL. a, Results for genes causally implicated in PD across cell types and activation states. Colocalisation defined by PPH4 > 0.75; Distinct is defined by PPH3 > 0.75; Suggestive is defined where the ratio of PPH4/PPH3 is high (ratio_PPH4_PPH3 > $\log_2(9)$) , but there is insufficient power to conclude a significant colocalisation (PPH3 + PPH4 is between 0.5-0.75). NS, no stimulation; IAV, influenza A virus; COV, SARS-CoV-2 virus).

Supplementary Figure 4

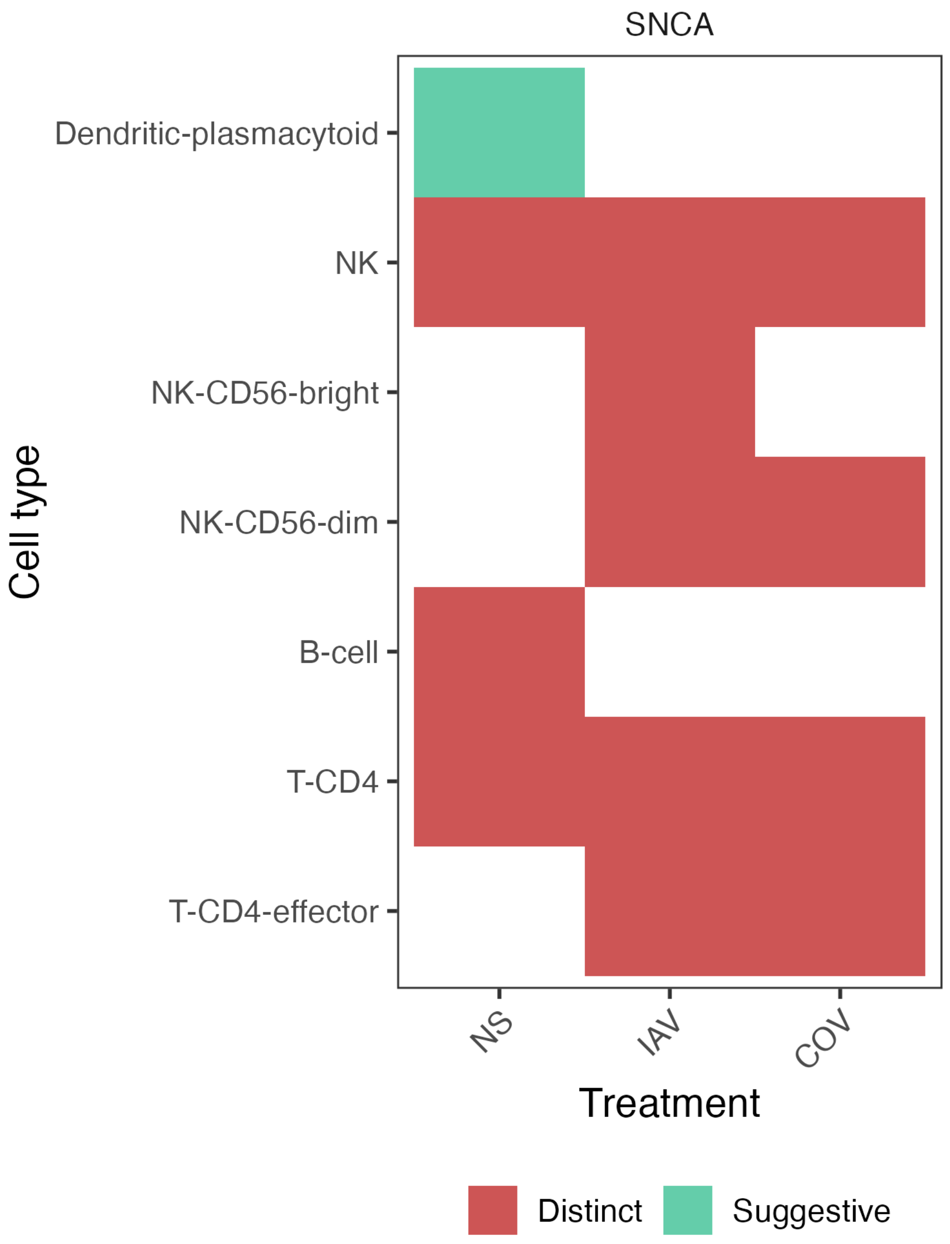

Supplementary Fig 4. Colocalisation results in East Asian PD GWAS and Multi-ancestry eQTL. a, Results for genes causally implicated in PD across cell types and activation states. Colocalisation defined by PPH4 > 0.75; Distinct is defined by PPH3 > 0.75; Suggestive is defined where the ratio of PPH4/PPH3 is high (ratio_PPH4_PPH3 > $\log_2(9)$) , but there is insufficient power to conclude a significant colocalisation (PPH3 + PPH4 is between 0.5-0.75). NS, no stimulation; IAV, influenza A virus; COV, SARS-CoV-2 virus.

Supplementary Figure 5

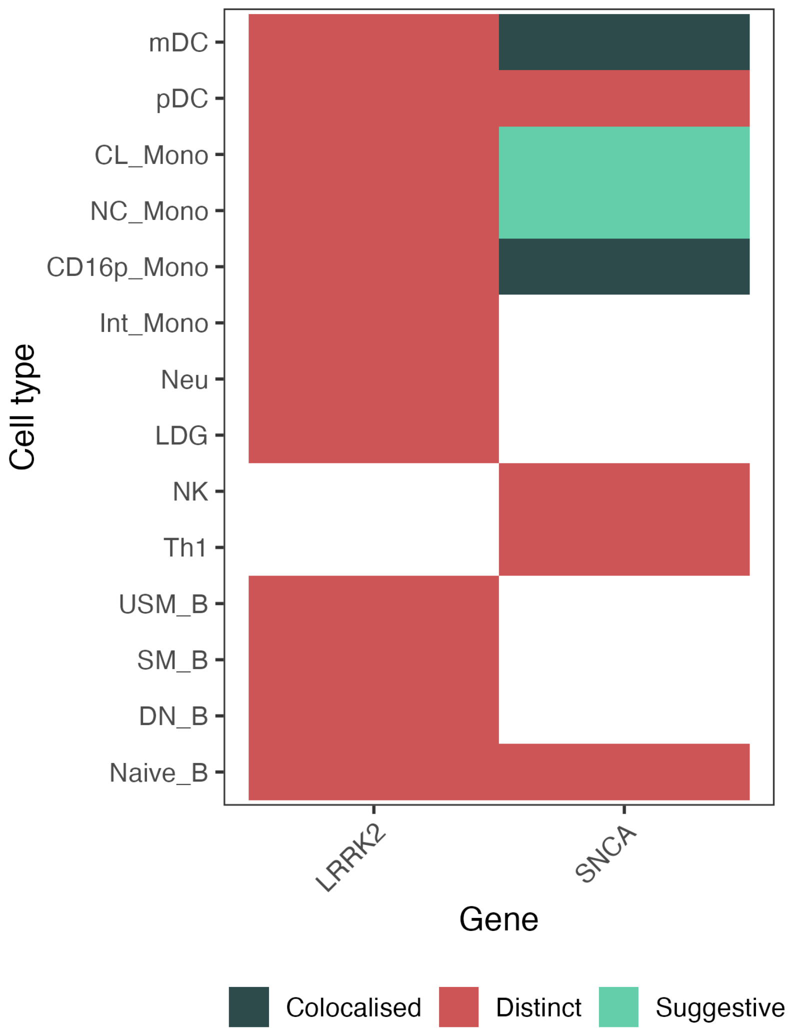

Supplementary Fig 5. Colocalisation results in East Asian PD GWAS and East Asian eQTL. Results for genes causally implicated in PD across cell types. Colocalisation defined by PPH4 > 0.75; Distinct is defined by PPH3 > 0.75; Suggestive is defined where the ratio of PPH4/PPH3 is high (ratio_PPH4_PPH3 > $\log_2(9)$), but there is insufficient power to conclude a significant colocalisation (PPH3 + PPH4 is between 0.5-0.75). NS, no stimulation; IAV, influenza A virus; COV, SARS-CoV-2 virus).

Supplementary Bibliography

1. Neuner, S. M., Tcw, J. & Goate, A. M. Genetic architecture of Alzheimer's disease. *Neurobiol. Dis.* **143**, 104976 (2020).
2. Blauwendraat, C., Nalls, M. A. & Singleton, A. B. The genetic architecture of Parkinson's disease. *Lancet Neurol.* **19**, 170–178 (2020).
3. Antonioni, A. *et al.* Frontotemporal Dementia, Where Do We Stand? A Narrative Review. *Int. J. Mol. Sci.* **24**, 11732 (2023).
4. Aquino, Y. *et al.* Dissecting human population variation in single-cell responses to SARS-CoV-2. *Nature* **621**, 120–128 (2023).
5. Chen, M.-H. *et al.* Trans-ethnic and Ancestry-Specific Blood-Cell Genetics in 746,667 Individuals from 5 Global Populations. *Cell* **182**, 1198-1213.e14 (2020).
6. Foo, J. N. *et al.* Identification of Risk Loci for Parkinson Disease in Asians and Comparison of Risk Between Asians and Europeans: A Genome-Wide Association Study. *JAMA Neurol.* **77**, 746–754 (2020).
7. Jansen, I. E. *et al.* Genome-wide meta-analysis identifies new loci and functional pathways influencing Alzheimer's disease risk. *Nat. Genet.* **51**, 404–413 (2019).
8. Nalls, M. A. *et al.* Identification of novel risk loci, causal insights, and heritable risk for Parkinson's disease: a meta-analysis of genome-wide association studies. *Lancet Neurol.* **18**, 1091–1102 (2019).
9. Nédélec, Y. *et al.* Genetic ancestry and natural selection drive population differences in immune responses to pathogens. *Cell* **167**, 657-669.e21 (2016).
10. Ota, M. *et al.* Dynamic landscape of immune cell-specific gene regulation in immune-mediated diseases. *Cell* **184**, 3006-3021.e17 (2021).
11. Quach, H. *et al.* Genetic adaptation and neandertal admixture shaped the immune system of human populations. *Cell* **167**, 643-656.e17 (2016).
12. Rizig, M. *et al.* Genome-wide Association Identifies Novel Etiological Insights Associated with Parkinson's Disease in African and African Admixed Populations. *medRxiv*

2023.05.05.23289529 (2023) doi:10.1101/2023.05.05.23289529.

13. Shigemizu, D. *et al.* Ethnic and trans-ethnic genome-wide association studies identify new loci influencing Japanese Alzheimer's disease risk. *Transl. Psychiatry* **11**, 1–10 (2021).
